# Epidemiologic Investigations into Outbreaks of Rift Valley Fever in Humans, South Africa, 2008–2011

**DOI:** 10.3201/eid1912.121527

**Published:** 2013-12

**Authors:** Brett N. Archer, Juno Thomas, Jacqueline Weyer, Ayanda Cengimbo, Dadja E. Landoh, Charlene Jacobs, Sindile Ntuli, Motshabi Modise, Moshe Mathonsi, Morton S. Mashishi, Patricia A. Leman, Chantel le Roux, Petrus Jansen van Vuren, Alan Kemp, Janusz T. Paweska, Lucille Blumberg

**Affiliations:** National Institute for Communicable Diseases–National Health Laboratory Service (NICD-NHLS), Johannesburg, South Africa (B.N. Archer, J. Thomas, J. Weyer, A. Cengimbo, C. Jacobs, S. Ntuli, P.A. Leman, C. le Roux, P. Jansen van Vuren, A. Kemp, J.T. Paweska, L. Blumberg);; University of the Witwatersrand, Johannesburg, South Africa (J. Thomas, L. Blumberg);; NICD-NHLS–School of Health Systems and Public Health, University of Pretoria, South Africa (D.E. Landoh, M. Modise, M. Mathonsi, M. S. Mashishi);; Ministère de la Santé, Lomé, Togo (D.E. Landoh).

**Keywords:** Rift Valley fever, Rift Valley fever virus, disease outbreak, zoonoses, South Africa, epidemiology, risk factors, occupational exposure, viruses

## Abstract

Rift Valley fever (RVF) is an emerging zoonosis posing a public health threat to humans in Africa. During sporadic RVF outbreaks in 2008–2009 and widespread epidemics in 2010–2011, 302 laboratory-confirmed human infections, including 25 deaths (case-fatality rate, 8%) were identified. Incidence peaked in late summer to early autumn each year, which coincided with incidence rate patterns in livestock. Most case-patients were adults (median age 43 years), men (262; 87%), who worked in farming, animal health or meat-related industries (83%). Most case-patients reported direct contact with animal tissues, blood, or other body fluids before onset of illness (89%); mosquitoes likely played a limited role in transmission of disease to humans. Close partnership with animal health and agriculture sectors allowed early recognition of human cases and appropriate preventive health messaging.

## Abstract 2

Rift Valley fever continues to pose a notable public health threat to humans.

Rift Valley fever (RVF) is an emerging arboviral zoonosis, endemic to Africa. During periods of anomalous heavy and prolonged rainfalls that favor the breeding of competent mosquito vectors, Rift Valley fever virus (RVFV) can cause widespread epidemics in livestock in the absence of high vaccination coverage. These large outbreaks are associated with high rates of abortion and death among domestic and wild ruminant animals. Not only do these outbreaks have a substantial socioeconomic effect, but they also pose a public health threat to humans (*1*,*2*).

Numerous routes of transmission of RVFV to humans have been observed during previous epizootics, with varying contributions to the overall epidemiologic profile. These routes include direct contact with infected animal tissues, blood, or other body fluids; inhalation of aerosolized infected fluids; and transmission through bites of infected mosquito vectors (*1*–*3*). Ingestion of raw and unpasteurized milk has also been epidemiologically associated with RVF disease in humans in previous outbreaks (*4*–*8*). A causal link between consumption of milk from infected animals and human infection has, however, not been conclusively demonstrated, and laboratory analysis of milk from experimentally infected animals provides conflicting evidence (*9*–*11*).

Because of the zoonotic nature of the virus, specific occupational groups are at increased risk of infection, such as farm, abattoir (slaughterhouse), veterinary, and allied animal-health workers (*1*–*3*,*12*,*13*). Most infections with RVFV in humans are asymptomatic or self-limiting, mild, influenza-like illness. However, in a small proportion of patients, severe complications can manifest as hemorrhage, encephalitis, hepatitis, or retinitis (*1*,*2*,*14*). The overall case-fatality rate is estimated to be 0.5%–2.0% (*1*).

Large epidemics were most recently documented in Somalia (2006–2007), Kenya (2006–2007), Tanzania (2007), Sudan (2007–2008), Mayotte (2007–2008), and Madagascar (2008) (*15*). Before 2008, South Africa experienced 2 large epizootics on the interior plateau (Free State, Eastern Cape, and Northern Cape provinces) during 1950–1951 (*16*) and again during 1974–1976 (*17*); however, smaller sporadic outbreaks have been regularly reported since the 1950s. We previously documented the reemergence of RVF in South Africa during 2008, when a cluster of veterinarians and animal farmers became ill after an outbreak among cattle on a dairy farm (*13*). The present study documents the investigation of human RVF cases observed from 2008 to 2011 and describes temporal and spatial trends, demographic characteristics, and exposure to RVFV.

## Case Detection and Outbreak Investigations

Following reports of RVF outbreaks in domestic and wild ruminants during 2008, systems for identifying and testing suspected RVFV infection in humans were enhanced. Throughout the study period (2008–2011), health care professionals were encouraged to consider RVF in the differential diagnosis of patients who met the suspected case criteria given below. Health care professionals were reached through various communication methods, including the distribution of guidelines (*18*) to health care facilities throughout the country, provision of regular reports and recommendations in newsletters of the National Institute for Communicable Diseases (NICD) that were widely distributed through health care profession networks, and numerous presentations in South Africa. Site visits and health promotional and enhanced case-finding activities were conducted by local health and veterinary authorities following reports of RVF in animal populations. Symptomatic persons identified during site visits were referred to local health care facilities for further assessment. A suspected RVF case-patient was defined as any person meeting  >1 of the following criteria: 1) a person belonging to a high risk category who has an influenza-like illness, which could include fever, myalgia, arthralgia, or headache; 2) a person belonging to a high risk category who has signs and symptoms of encephalitis, such as hemorrhage, hepatitis, or ocular pathology/retinitis, with or without fever; or 3) a person with unexplained encephalitis, hepatitis, or hemorrhagic illness who resides in an area where RVF can potentially occur. High risk categories included the following: a) recent close contact with livestock and game animals in or from RVF-affected areas, including slaughtering and butchering (traditional or commercial), disposal of carcasses and fetuses, assisting with birthing or other animal husbandry activities that resulted in exposure to animal blood and body fluids, or veterinary procedures and necropsies; b) residing in an area where RVF is known to occur or has the potential to occur and recent mosquito bites; or c) consuming unpasteurized milk from RVF-affected areas.

Two clotted blood specimens were requested from all persons who met the suspected case definition. Specimens were transported to the Special Pathogens Unit, NICD, where assays were performed in order to detect RVFV-specific nucleic acid and antibodies against RVFV. These assays included a combination of real-time reverse transcription PCR, loop-mediated isothermal amplification assays, virus isolation, hemagglutination-inhibition assays, or IgM ELISA, as per previously published protocols (*19*–*21*). We also considered cases identified through specimens submitted for routine arbovirus testing. A confirmed case was defined as the detection of live RVFV, RNA, or IGM against-RVF.

Basic patient identification and demographic information accompanied specimens. After identifying a confirmed case, we interviewed the attending health care worker and patient (when possible) to complete a standardized questionnaire. In most instances, interviews were completed by telephone, but occasionally interviews were conducted during field visits. The questionnaire captured the following: demographic details, including patient’s age, sex, address and location, and occupation , clinical details, including timing of illness onset, symptoms, sequelae, clinical outcome, hospital admissions, and past medical history (clinical findings to be presented elsewhere); and exposure details. For the latter, we asked whether the case-patient had experienced any of the following exposure categories in the week before illness onset: contact with animal tissues, blood, or body fluid; mosquito bites; drinking unpasteurized milk; or, acquiring, handling, or consuming meat either directly from a farm or from an informal or traditional butcher. These questions were not mutually exclusive, and case-patients could report experiencing >1 exposure category. The questionnaire additionally allowed for comments to describe details about recalled exposure events, which were later coded for data capturing and analysis.

Data from specimen submission records, laboratory reports, and questionnaires were captured, combined, cleaned, and analyzed in a combination of Excel 2003 (Microsoft, Redmond, WA, USA) and EpiInfo v3.5.3 (Centers for Disease Control and Prevention, Atlanta, GA, USA). In this study, we considered all persons with a completed laboratory investigation for RVF in South Africa from 2008 to 2011 and focused subanalyses on confirmed cases only. When date of symptom onset was not available, we estimated the date on the basis of the date of specimen collection. Incidence rates (IR) were calculated using the annual Statistics South Africa midyear population estimates for 2008–2011 (*22*). Spatial analyses were completed in ArcGIS v10.0 (ESRI, Redlands, CA, USA). We geocoded confirmed cases to an administrative local municipality using an address or nearest reported town. The revised South African municipal boundaries, released June 28, 2011, were used for spatial analysis for all years (*23*).

Ethics clearance for essential communicable disease surveillance was granted to the NICD by the Human Medical Research Ethics Committee of the University of the Witwatersrand, Johannesburg (protocol number M060449, reference R14/49 Schoub). This clearance includes outbreak investigations related to notifiable medical conditions under surveillance, including RVF.

## Temporal and Spatial Trends

A total of 2,621 specimens were tested for RVFV from 2008 through 2011. We excluded duplicated sets of specimens from patients tested on >2 occasions (n = 93), specimens collected from patients outside of South Africa (n = 6), and specimens from those who did not meet the suspected case definition and tested negative for RVFV infection (n = 513). Of the remaining 2,009 suspected cases, 302 cases were laboratory-confirmed (15% detection rate). Interviews were completed for 245 (84%) confirmed cases; however, partial descriptive data were available through specimen submission records for all confirmed cases, and therefore, we considered all confirmed cases in further analyses.

**Figure 1:**
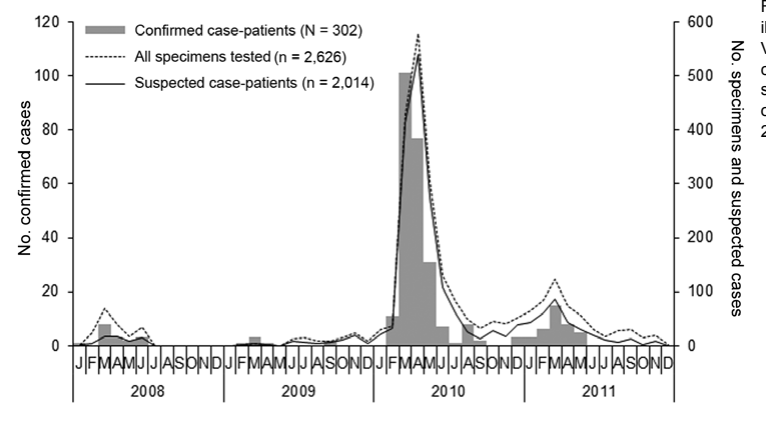
Epidemic curve illustrating the frequency of Rift Valley fever laboratory-confirmed cases, all specimens tested, and suspected cases tested by month of illness onset, South Africa, 2008–2011 (N = 302).

Following the reemergence of RVF in 2008, outbreaks of the disease were noted for 4 consecutive years, and the influx of specimens and incidence of confirmed human cases in late summer to early autumn months increased annually and peaked in March each year (Figure 1). Few or no cases were detected during the colder winter months of each year.

**Figure 2:**
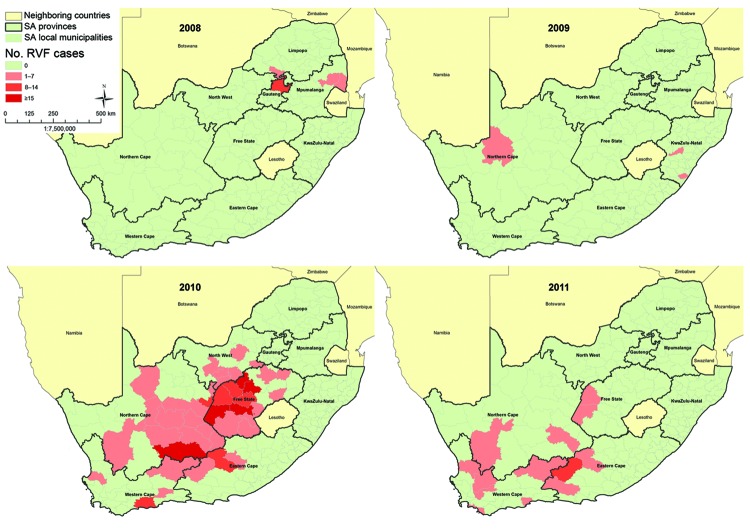
The spatial frequency distribution of human laboratory-confirmed Rift Valley fever cases by administrative local municipality by year, South Africa (SA), 2008–2011 (N = 302).

Sporadic RVF outbreaks, of relatively limited spatial extent and magnitude, were observed during 2008 and 2009. A total of 17 confirmed cases (IR 0.03/100,000 persons) were detected during 2008 from 3 of the 4 provinces that reported RVF among animal populations at that time (Table 1, Figure 2). During 2009, a total of 7 confirmed cases (IR 0.01/100,000 persons) were detected following isolated, sporadic outbreaks among animals in KwaZulu-Natal and Northern Cape provinces.

**Table 1:** Frequency and incidence rate of human laboratory-confirmed Rift Valley fever cases by province where exposed, stratified by year, South Africa, 2008–2011*

Province	2008			2009			2010			2011			Total
	No. (%)	IR		No. (%)	IR		No. (%)	IR		No. (%)	IR		No. (%)
Free State	0	0		0	0		125 (52)	4.43		3 (8)	0.11		128 (43)
Northern Cape	0	0		2 (29)	0.17		80 (33)	7.25		3 (8)	0.27		85 (28)
Eastern Cape	0	0		0	0		16 (7)	0.24		17 (46)	0.25		33 (11)
Western Cape	0	0		0	0		11 (5)	0.21		14 (38)	0.26		25 (8)
Gauteng	9 (53)	0.09		0	0		0	0		0	0		9 (3)
North West	0	0		0	0		8 (3)	0.25		0	0		8 (3)
Mpumalanga	6 (35)	0.17		0	0		0	0		0	0		6 (2)
KwaZulu-Natal	0	0		5 (71)	0.05		0	0		0	0		5 (2)
Limpopo	2 (12)	0.04		0	0		0	0		0	0		2 (1)
Total	17	0.03		7	0.01		240*	0.48		37	0.07		301†
*N = 302; IR, incidence rate/1000,000 persons. †Province known for 301 (99%) cases; province data not available for 1 case-patient in 2010.													

During February 2010, following heavy rainfalls across large parts of the country, an explosive and geographically extensive RVF epizootic occurred (Figures 1, 2). A total of 241 confirmed cases were identified. The outbreak was most concentrated in the interior plateau of South Africa, and the highest rates of human infection were observed in Northern Cape (IR 7.25/100,000 persons) and Free State (IR 4.43/100,000 persons) provinces.

Human RVF cases continued to be detected from December 2010 to May 2011; most were diagnosed in areas bordering Eastern Cape and Western Cape provinces. Thirty-seven human cases were confirmed (IR 0.07/100,000 persons) in 2011. The last confirmed human RVF case reported illness onset on May 21, 2011.

## Demographic Description

Table 2 gives a demographic description of the 302 confirmed cases. The ratio of male to female cases was 6.55 to 1, and infection was predominantly among adults (median age 43 years, range 1 to 86 years). Data on occupation were available for 289 (96%) of persons with confirmed cases. Of these, animal farmers and animal farm workers (n = 173, 60%) made up the largest proportion. Infection among persons working in various animal health and veterinary science professions (n = 37, 13%) and persons in a meat-related industry (n = 32, 11%) were also frequently reported. Collectively, 242 (83%) persons with confirmed cases reported working within occupations in which direct contact with animals frequently occurs.

**Table 2:** Frequency distribution of human laboratory-confirmed Rift Valley fever cases by patient characteristic, South Africa, 2008–2011

Characteristic	No. (%); N = 302
Male sex*	262 (87)
Age group, y†	
0–9	1 (<1
10–19	16 (5)
20–29	67 (22)
30–39	47 (16)
40–49	68 (23)
50–59	53 (18)
60–69	30 (10)
≥70	18 (6)
Occupation‡	
Farmer or farm worker	173 (60)
Animal health worker	37 (13)
Abattoir worker, butcher, or hunter	32 (11)
Farm resident (nonworker)	5 (2)
Non–animal related occupation	42 (15)
*Known for 302 case-patients. †Known for 300 case-patients. ‡Known for 289 case-patients	

Twenty-five of the confirmed case-patients died from RVF (overall CFR, 8%). All deaths occurred during 2010 (year-specific CFR, 10%). The median age at illness onset among patients with fatal cases was 47 years (range, 15–72 years).

## Exposures

Individual exposure history before onset of symptoms was obtained from 284 (94%) confirmed case-patients. Of these, 254 (89%) reported a history of direct contact with animal tissues, blood, or body fluid; 46 (16%) noted being bitten by mosquitoes; 30 (11%) reported drinking unpasteurized milk; and 21 (7%) reported acquiring, handling, or consuming meat directly from a farm or an informal or traditional butcher.

In addition, we considered the frequency of each exposure classification among case-patients reporting only a single event. Of 234 case-patients who met these criteria, 205 (88%) reported only a history of direct contact with animals, 15 (6%) reported only being bitten by mosquitoes, 6 (2%) reported only drinking unpasteurized milk, 3 (1%) reported only acquiring, handling, or consuming meat either directly from a farm or informal or traditional butcher, and 5 (2%) could not recall any of the listed exposure types.

Of 254 case-patients who reported a history of direct contact with animals, 169 (67%) provided comments that allowed further categorization of animal-related exposures before onset of illness. Comments were open-ended, and >1 activity was often reported by a single case-patient. Of the 169 case-patients who provided additional comments, 136 (80%) reported physical contact with animal carcasses, either during the disposal of animals that died of RVF or during other procedures. Slaughtering of livestock or game animals was documented in 70 (41%) cases, and included reports of animal slaughter on farms (n = 35), in commercial abattoirs (n = 26), while hunting (n = 4), and in unspecified locations (n = 5). The performance of necropsies on animals was documented in 32 (19%) cases, including 26 instances in which necropsies were performed by animal health professionals, and 6 instances in which necropsies were undertaken by animal farmers or animal farm workers. Handling and disposing of fetal material after abortions in pregnant ruminants was documented for 28 (16%) cases. Exposures were, however, not limited to deceased animals but also included physically assisting with the birthing of live animals (n = 16; 9%), unspecified veterinary procedures (n = 8, 4%), and shearing (n = 2, 1%). Eighteen (11%) case-patients reported vaccinating livestock against RVF; of these, 2 case-patients reported needle-stick injuries while administering vaccine.

## Conclusions

During sporadic RVF outbreaks in 2008–2009 and extensive epidemics during 2010–2011, a total of 302 laboratory-confirmed human infections were identified. The incidence of human cases peaked in the late summer to early autumn months of each year and was spatially concentrated in the inferior plateau, later extending down to the southern coastal provinces of South Africa. This coincided with epizootics observed following heavy rainfall, and we observed spatial and temporal patterns for human RVF infections similar to those observed in RVF outbreaks reported in domestic livestock (*24*). The sporadic cases in 2008–2009 were attributed to RVFV lineage C, which is distributed widely and has been responsible for epizootics throughout Africa and the Arabian Peninsula (*25*). RVFV lineage H, an apparent antecedent from Namibia in 2004, was responsible for the 2010–2011 South African epizootics (*25*). A degree of spatial overlap was observed when comparing outbreaks in 2010 to 2011; however, in 2011, human infections were primarily observed in areas that were not previously affected. This finding may be explained by accumulated herd immunity in areas affected in 2010, attributable to a combination of natural infections and extensive vaccinations conducted in livestock populations, while neighboring populations remained susceptible to RVFV outbreaks in 2011. Two livestock vaccines were applied during the 2008–2011 outbreaks: inactivated whole RVFV vaccine, which requires a booster vaccination and annual revaccination; and the live-attenuated Smithburn vaccine, which provides lifelong immunity but may cause abortions and fetal malformations when administered to gestating adult animals.

Laboratory-confirmed human cases were typically in men who worked in animal farming, animal health, and meat-related industries. Most (89%) case-patients reported direct contact with animal tissues, blood, or other body fluids, which suggests that this is the most common risk factor and route of transmission to humans in South Africa. Slaughtering animals in commercial and farm settings, conducting necropsies, and handling fetuses of aborted ruminants were frequently documented as specific exposure events. These findings are consistent with risk factor analyses conducted during the 2007 RVF outbreaks in Kenya and Tanzania, in which ≈40% of cases had direct contact with sick animals (*3*,*7*). In the South African outbreaks reported here, however, the role of transmission through direct contact appears to have been even more predominant. During the 2000–2001 outbreaks in Saudi Arabia, researchers showed that in 23% of cases transmission likely occurred through mosquito exposure (*26*). Although we observed that mosquito vectors played a role in establishing RVF in South Africa and amplifying the epizootic among animal populations, this route appears to have a limited role in transmission of RVFV to humans (16% of case-patients reported mosquito bites; 6%reported mosquito bites as the only exposure). Similarly, other possible routes of transmission, such as consuming fresh, unpasteurized milk likely played a lesser role in human infections in South Africa. Indeed, ingesting unpasteurized milk has only been implicated epidemiologically (*4*–*8*); nonetheless, it remains a consideration for health promotional interventions.

Exposure to RVFV during animal vaccinations was the likely route of transmission in a limited number of cases. As livestock vaccines are commonly sold in multidose vials and are administered with automatic syringes with intermittent needle changes, vaccine may inadvertently be given to viremic livestock, with the potential for serial transfer of wild RVFV to other animals or to humans through needle-stick injuries. Reassortant RVFVwas identified in a patient who experienced a needle-stick injury and was potentially exposed to both live vaccine and wild virus supports this assumption (*25*).

We found an overall CFR of 8% for all laboratory-confirmed RVF cases identified in 2008–2011. This rate is substantially higher than the generally accepted death rate (0.5%–2.0%) but lower than that observed in Saudi Arabia (13.9% of confirmed cases), Kenya (26.5%) and Tanzania (47%) (*1*,*7*,*26*,*27*). However, in many RVF outbreak investigations (as in this study), the most clinically severe cases are often those that are detected and confirmed.

Our investigation was limited in its capacity to detect asymptomatic and subclinical RVFV infections. Serologic surveys conducted following outbreaks elsewhere have found most human RVF case-patients remain asymptomatic after infection (*1*). Recall bias may have also affected our findings because of delays incurred between onset of illness, laboratory-confirmation of RVF infection, and completion of interviews. Noteworthy events, such as animal birthing or slaughtering, may have been more easily recalled by patients. Likewise, in some instances interviews were limited to only the clinician treating the patients, who would likely rely on information recorded in a patient’s file.

Despite these limitations, this study provides a reliable minimum estimate of the magnitude of human infections during 4 years of heightened RVFV transmission among animal populations. The availability of extensive laboratory capacity for testing all suspected RVF cases afforded a unique opportunity (both for South Africa and the African continent) to support RVF outbreak investigations on such a scale. Close collaboration between field investigators and the laboratory is vital, given the nonspecific nature of mild RVF disease in humans, which can be easily confused with diverse causes of febrile illnesses. The combination of multiple laboratory assays for direct detection of RVFV, as well as serologic evidence of acute infection, proved successful in identifying cases. Equally important was a close working relationship between health and agriculture sectors, which illustrates the importance of intersectoral responses to zoonotic outbreaks and a successful One Health Initiative approach. In previous outbreaks, detecting severe (usually hemorrhagic) illness in humans was often the catalyst for investigations. In the present study, our investigations were triggered in response to surveillance conducted by agriculture authorities; these early warnings also provided an opportunity for preventative interventions and timely response to human RVF cases in many instances.

When will the next RVF epizootic occur in South Africa? La Niña weather conditions with heavy rain falls were predicted for the early months of 2012; however, further RVF outbreaks were not observed. Extensive livestock vaccination against RVFV during recent years may have resulted in decreased susceptibility of the host populations, reducing the likelihood of further explosive epidemics in the short term. However, we may be entering another interepizootic period of unknown duration. The natural history of the disease in South Africa has been in part attributable to animal vaccination practice; epidemics prompt mass vaccination of livestock, followed by a dramatic drop-off in vaccination coverage rates during extended interepizootic periods, which leads to the accumulation of large numbers of susceptible animals that are not immune and sets the stage for explosive epidemics in concert with conducive climatic conditions. Ongoing preparedness and continued preventative interventions aimed towards population groups at high risk and practices identified by this study will be essential in reducing the effect of future epizootics on human populations. Continued strengthening of surveillance studies in humans, livestock, and wild animals will be critical in enabling a rapid response to RVF epidemics, as well as preventing future widespread epizootics.
